# The Impact of Sleeve Gastrectomy Combined With Lifestyle Interventions on Anthropometric and Health Outcomes in Adults: A Systematic Review and Meta-Analysis

**DOI:** 10.7759/cureus.85587

**Published:** 2025-06-09

**Authors:** Anees A Alyafei, Said K AlMukhaini, Aysha MA Hussein, Sara Tariq Al Abdulla

**Affiliations:** 1 Preventive Medicine/Wellness Programs, Primary Health Care Corporation, Doha, QAT; 2 Health Services and Programs, Ministry of Health, Muscat, OMN; 3 Wellness Programs, Preventive Health, Primary Health Care Corporation, Doha, QAT

**Keywords:** bariatric surgery', lifestyle interventions, obesity, post-sleeve gastrectomy, previous sleeve gastrectomy, wearable devices, weight loss and obesity

## Abstract

Weight loss from obesity is a global health concern associated with increased risk of chronic illness and significant healthcare costs. Sleeve gastrectomy is an increasingly popular bariatric surgery option for substantial weight reduction; however, sustained success depends on lifestyle modification. Integrating food, exercise, behavioral, and technology-based interventions into surgery can potentially enhance outcomes and promote weight maintenance. A systematic review and meta-analysis were conducted in accordance with the Preferred Reporting Items for Systematic Review and Meta-analyses (PRISMA) 2020 guidelines, encompassing randomized controlled trials published between 2014 and 2025. Databases like PubMed, Cochrane Library, Embase, Web of Science, and Scopus were searched. Studies included adults (age ≥18 years) with obesity undergoing sleeve gastrectomy and subjected to post-operative lifestyle interventions. The primary outcomes were weight changes, BMI, and body composition, while the secondary outcomes included comorbidities and quality of life. Risk of bias was assessed using the ROB-2 tool (www.cochrane.org). Thirty-one studies with varying follow-up durations (1-60 months) were included. The combined intervention resulted in greater weight loss (5%-30% decrease), improvement in fat mass, BMI, and cardiometabolic profiles compared to usual care. The meta-analysis revealed a high overall effect size for weight reduction, with a value of 2.56 (2.15-2.88) for the high weight reduction group and 1.88 (1.76-1.91) for the low weight reduction group. Heterogeneity among studies was moderate, with I² values ranging from 18% to 46%, indicating some variation in study populations and intervention effects. Technology-augmented interventions, such as wearable devices and mobile apps, provided additional gains in adherence and long-term efficacy. Long-term weight loss maintenance remained a difficulty. Sleeve gastrectomy combined with structured lifestyle interventions significantly improves anthropometric and health outcomes in adults. Technology-derived interventions and behavioral therapy have the potential for improving long-term weight management, but additional studies are required to optimize intervention approaches.

## Introduction and background

Obesity has become one of the most significant health issues of the 21st century, with its occurrence spreading at a very rapid rate across the globe [1]. According to the World Health Organization (WHO), the rate of obesity has tripled since 1975, and in 2016, over 1.9 billion adults were overweight and 650 million were obese. In 2022, global obesity rates were alarmingly high, with one in eight people living with obesity. Adult obesity has more than doubled since 1990, while adolescent obesity has quadrupled. That year, 2.5 billion adults (aged 18 years and older) were overweight, with 890 million of them living with obesity. Among adults, 43% were overweight and 16% were living with obesity. Childhood obesity also remains a significant concern, with 37 million children under the age of five years being overweight, and over 390 million children and adolescents aged 5-19 were overweight, including 160 million living with obesity [2]. This condition also puts one at a higher risk of developing many chronic diseases, such as type 2 diabetes, cardiovascular disease, cancers, and sleep apnea. Aside from physical health, obesity has also been linked to a variety of mental illnesses such as depression, anxiety, and low self-esteem. Obesity also costs the healthcare systems heavily because the treatment of obesity complications is very costly [3,4].

Bariatric surgery is now employed as an effective and common treatment in patients with complex obesity who have been unable to obtain satisfactory weight loss using conventional methods of diet and exercise [5,6]. Among the various bariatric operations, sleeve gastrectomy (SG) has become a much-appreciated intervention due to its proven efficacy, relatively low complication rate, and reduced post-operative care requirement. SG involves the resection of the majority of the stomach, resulting in an 80% reduction in size, with a residual tube-like structure remaining [7,8]. It has been said to result in dramatic weight loss and improvement of comorbidities in conditions such as type 2 diabetes mellitus, hypertension, and dyslipidemia. Furthermore, SG is associated with lower morbidity and mortality rates compared to other types of bariatric surgery, making it an attractive option for both patients and doctors [9].

While sleeve gastrectomy has been remarkably successful in creating weight loss, long-term success depends significantly on acquiring and maintaining lifestyle changes [10,11]. Individuals who undergo surgery after it have to embrace a multidisciplinary method of managing weight, including dietary change, increased physical activity, and behavior therapy. These lifestyle changes play a key role in preventing weight regain, which is the most common issue in long-term follow-up among patients [12]. Post-surgery diets typically consist of frequent, small meals that are high in protein and low in sugar [13,14]. Increasing physical activity is crucial, as exercise promotes energy expenditure, improves cardiovascular health, and preserves lean body mass. In addition, behavioral therapies such as cognitive-behavioral therapy, self-monitoring, and goal setting can significantly promote patients' adherence to weight loss programs and facilitate their maintenance of the necessary behavioral changes that result in long-term weight management [13,15].

Advances in wearable technology and mobile health (mHealth) applications have provided new ways of supporting patients in adhering to these lifestyle changes [16]. Wearable technology has tracked physical activity, dietary consumption, and even nocturnal activity, and these devices have become widely used aids for weight loss [17,18]. Technology-based interventions provide real-time feedback, tailored guidance, and motivation, encouraging patients to remain active in their postoperative treatment. Despite an increase in interest in these technologies, evidence of their influence when applied as an adjunct to sleeve gastrectomy is slim, with various studies approximating the impact of such interventions at best as minimal. Thus, A literature review should determine the combined effects of technology-based lifestyle intervention and sleeve gastrectomy [19,20].

This study is significant since it attempts to bridge the gap in the literature on the application of technology-based interventions to post-surgical weight control following sleeve gastrectomy. While sleeve gastrectomy has been proven to result in weight loss, there is no consensus on whether lifestyle interventions, particularly those that utilize technology, have a lasting impact on enhancing post-surgical outcomes [21]. Mobile applications and wearable devices as part of post-operative care hold promise. However, their ability to support long-term weight loss and improved health outcomes is not yet fully established. By systematically examining and synthesizing existing knowledge, this study aims to determine how incorporating sleeve gastrectomy with lifestyle interventions, particularly technology-based ones, can enhance long-term patient outcomes [22,23].

The primary purpose of this review is to assess the impact of sleeve gastrectomy combined with lifestyle interventions on the health and anthropometric measures in adults. This includes examining the effects of diet, exercise, and behavioral change, as well as those of wearable devices and mobile applications, on weight reduction, the management of obesity comorbidities, and body composition. In addition to these primary outcomes, the study will also assess secondary outcomes, including the effects of these interventions on patients' psychology and quality of life, as well as sustained weight loss over a long duration after surgery. In accomplishing such goals, the study is most likely to yield extensive information on the use of lifestyle and technology interventions in the long-term success of sleeve gastrectomy. Lastly, this review will provide helpful information on the synergistic effects of sleeve gastrectomy and lifestyle interventions, such as those utilizing technology, in achieving optimal weight loss outcomes and managing obesity-related disorders. The findings of this research will inform clinical practice and identify areas of highest research priority for future studies, thereby bridging the gap between surgical weight loss interventions and long-term weight maintenance interventions.

## Review

Methodology

The meta-analysis and systematic review assessed the overall effects of lifestyle interventions and sleeve gastrectomy on anthropometric and health outcomes in adults. The review followed the PRISMA 2020 guidelines to enhance transparency and reliability [24]. This study included randomized controlled trials (RCTs) that compared the combined effects of sleeve gastrectomy with and without lifestyle interventions, such as dietary modifications, exercise, behavior change, and technology-based interventions in the form of wearable monitors or cell phone applications. PICO format (Population, Intervention, Comparison, Outcome) organized the research question and the analysis (Table 1).

The primary objective was to evaluate the impact on weight loss, body composition, and obesity-related comorbidities. The econdary objective was to assess the effect of intervention type, body composition changes, and follow-up duration on health outcomes by analyzing subgroup effect sizes. To evaluate the effectiveness of wearable technology in enhancing adherence and sustaining postoperative weight loss over 12-24 months. The protocol was pre-registered in the International Prospective Register of Systematic Reviews (PROSPERO) database with registration number CRD420251041610.

**Table 1:** The PICO framework for the impacts of lifestyle interventions and sleeve gastrostomy on anthropometric and health outcomes among adults PICO: Population, Intervention, Intervention, Comparison, Outcome; BMI: Body Mass Index.

PICO Element	Description
Population	Adults aged 18 years or older with obesity underwent sleeve gastrectomy and participated in post-surgical lifestyle interventions (diet, exercise, behavioral therapy, technology-based interventions).
Intervention	Sleeve gastrectomy combined with lifestyle interventions such as dietary modifications, physical activity, behavioral therapy, and/or wearable devices or mobile health applications.
Comparison	Sleeve gastrectomy combined with lifestyle interventions vs. no intervention or standard post-surgical care (e.g., routine follow-up without additional lifestyle interventions).
Outcomes	Weight loss (percentage of excess weight loss, total body weight loss, BMI reduction), devices

Eligibility criteria

We included RCTs that explored the concurrent influence of sleeve gastrectomy and lifestyle therapy among adults ≥18 years. Participants must have undergone sleeve gastrectomy and received post-surgical lifestyle interventions, including dietary modification, physical activity, behavioral therapy, or technology-based interventions such as wearable devices or mobile health applications. Studies should have provided at least one of the specified relevant outcomes, including weight loss, body composition changes, or obesity-related comorbidities (e.g., diabetes, hypertension, or dyslipidemia). We only utilized studies published between 2014 and 2025 to obtain the most recent evidence. Non-randomized trials, observational studies, and controlled studies without a control group were excluded from the analysis. Non-surgical interventions or reporting non-relevant outcomes were excluded. We excluded studies published in languages other than English unless they had a reliable English translation.

Search strategy

A systematic literature search was conducted in PubMed, Cochrane Library, Web of Science, Embase, and Scopus, with a cutoff date of January 1, 2014, to 2025. Studies that were selected based on predefined inclusion and exclusion criteria. The search utilized relevant keywords related to sleeve gastrectomy, bariatric surgery, weight loss, lifestyle interventions, and technology-based interventions, along with Boolean operators (OR, AND). Filters for ‘Randomized Controlled Trial’ were applied across all databases to restrict the results to RCTs only. Hand searches of the reference lists in the included articles and pertinent reviews were also conducted to identify other relevant studies. The grey literature, including conference abstracts and unpublished reports, was included if it provided substantial data. Two independent reviewers conducted the literature search and screened titles, abstracts, and full texts based on predefined eligibility criteria to ensure comprehensive identification and inclusion of relevant studies. Details of the search strategy are found in the Appendix.

Data extraction and management

Data extraction was independently carried out by two reviewers using a pre-formatted form [25]. The extraction form was pilot-tested on a subset of five studies and refined to ensure clarity, consistency, and completeness before full implementation. Discrepancies were resolved through consensus, and a third reviewer was consulted if necessary. Key variables extracted included study details (e.g., author, year, study design), participant demographics (e.g., sample size, gender, body mass index), intervention specifics (e.g., type of intervention, duration), and outcomes assessed (e.g., weight loss).

Primary outcomes of interest included weight loss, measured as percentage excess weight loss, total body weight reduction, or reduction in body mass index. The secondary outcomes included the decrease in obesity comorbidities (e.g., blood pressure). Data were stored in a standard spreadsheet for analysis using Microsoft Excel version 2023. EndNote reference management software was used to manage citations, and the extracted data were verified for accuracy.

Risk of bias assessment

Risk of bias in included studies was assessed by using the Risk of Bias (ROB)-2 (www.cochrane.org) tool for RCTs [26]. This tool measures bias across several domains, including the randomization process, deviation from intended interventions, missing outcome data, outcome assessment, and reporting of results. Each domain was rated as "low risk," "some concerns," or "high risk." Individual domain ratings were used to calculate the overall risk of bias. Two reviewers independently performed the assessment; disagreements were addressed through discussion or a third reviewer. Risk of bias assessment was significant in interpreting the results, as high-risk of bias studies might overestimate the effect of the intervention. Sensitivity analyses were conducted to assess the impact of excluding high-risk studies on the overall results.

Statistical methods

A random-effects model was used for meta-analysis to pool data between studies. The model is appropriate for use when there are heterogeneous studies regarding interventions, population characteristics, and outcomes [26]. Effect sizes for continuous outcomes (e.g., weight loss, body composition) were calculated as standardized mean differences (SMDs) with 95% confidence intervals (CIs). For dichotomous outcomes (e.g., comorbidity resolution), the effect size was presented as odds ratios (ORs) with 95% CIs. Heterogeneity across studies was assessed using the I² statistic, which estimates the proportion of variability across studies due to heterogeneity rather than chance. An I² value > 50% suggests moderate to high heterogeneity [27]. Statistical analyses were conducted using Review Manager (RevMan) version 5.4.1 (www.revman.cochrane.org).

Subgroup analysis

Subgroup analyses were also performed to examine the impact of specific factors on outcomes. These analyses included gender-based comparisons, the type of lifestyle intervention (e.g., diet, exercise, behavioral therapy, or technology-based interventions), and whether wearable devices or other technology-based aids were used. These analyses examined whether specific interventions or demographic variables influenced the effectiveness of sleeve gastrectomy combined with lifestyle interventions.

Results

Study Selection and Characteristics

**Figure 1:**
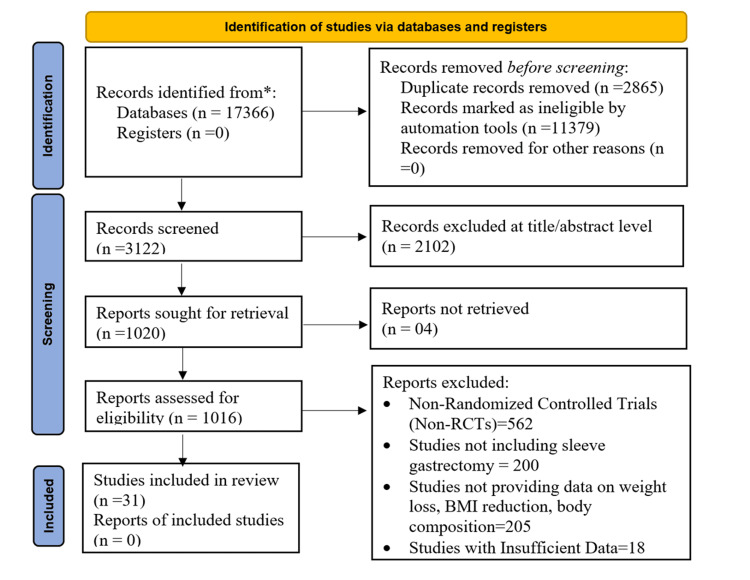
Study selection flow diagram based on PRISMA 2020 guidelines n: total number, PRISMA: Preferred Reporting Items for Systematic Reviews and Meta-Analyses; RCTs: Randomized Controlled Trials; BMI: Body Mass Index.

The PRISMA 2020 flow diagram presents the systematic selection of studies involved in the review on the Impact of Sleeve Gastrectomy Combined with Lifestyle Interventions on Anthropometric and Health Outcomes in Adults. Identification starts with 17,366 records accessed through databases, excluding duplicates and ineligible records by default. After screening 3,122 records, 2,102 were excluded based on title and abstract, and 1,016 reports were screened for eligibility. Out of these, 31 studies were ultimately shortlisted for review, ruling out non-randomized trials, sleeve gastrectomy trials, or trials with no valuable data on weight reduction, decrease in BMI, and body composition (Figure 1). Selected studies were characterized predominantly include individuals who have undergone bariatric surgery, particularly sleeve gastrectomy, and have incorporated lifestyle interventions such as exercise, dietary changes, and the use of wearable devices to monitor progress.

The significant results show fluctuations in the percentage of weight loss across the studies, ranging from 5% to a 30% reduction in body mass. Results also indicate increased differences in body composition, including a decrease in fat mass and an increase in lean mass gain. In terms of interventions, various exercise programs (e.g., high-intensity interval training, aerobic exercise) and nutritional interventions (e.g., Mediterranean diet, low-calorie diet) were added to sleeve gastrectomy. They demonstrated substantial improvements in physical health indicators, including a reduced BMI and waist circumference, as well as enhanced insulin sensitivity. Furthermore, wearable devices such as fitness watches, smart bathroom scales, and blood pressure monitoring were employed regularly to track health indicators and adherence to the interventions. The duration of follow-up in the studies ranged from one to five years, with studies showing improvements in various anthropometric and health indicators. For example, weight loss was consistently reported in most trials, with substantial evidence showing improved physical function, lowered blood pressure, and enhanced psychological well-being. Trials involving wearable technology often tracked real-time activity, weight loss, and even metabolic markers, such as glucose and lipid profiles. Overall, the results from the table underscore the importance of incorporating sleeve gastrectomy into lifestyle interventions to enhance weight control, body composition, and overall health outcomes in adults (Table 2).

**Table 2:** Characteristics of included studies BES: Binge Eating Scale; BMD: Bone Mineral Density; BMI: Body Mass Index; CBT: Cognitive Behavioral Therapy; CGM: Continuous Glucose Monitor; CON: Control Group; ECG: Electrocardiogram; EBL: Excess Body Loss; EES: Emotional Eating Scale; EWL: Excess Weight Loss; ESG: Endoscopic Sleeve Gastroplasty; FFM: Fat-Free Mass; FGF21: Fibroblast Growth Factor 21; GAD-7: Generalized Anxiety Disorder 7-item scale; GDF15: Growth Differentiation Factor 15; GLP-1RA: Glucagon-Like Peptide-1 Receptor Agonist; HbA1c: Hemoglobin A1c; HAPA: Health Action Process Approach; HDL: High-Density Lipoprotein; HF: High Frequency; HIIT: High-Intensity Interval Training; HRQoL: Health-Related Quality of Life; HRV: Heart Rate Variability; ILI: Intensive Lifestyle Intervention; LAGB: Laparoscopic Adjustable Gastric Banding; LF: Low Frequency; LWLI: Lifestyle Weight Loss Intervention; mHealth: Mobile Health; MICT: Moderate-Intensity Continuous Training; MLI: Multidisciplinary Lifestyle Intervention; MBS: Metabolic and Bariatric Surgery; MVPA: Moderate-to-Vigorous Physical Activity; NASH: Non-Alcoholic Steatohepatitis; NAFLD: Non-Alcoholic Fatty Liver Disease; PELI: Psychotherapy-Enhanced Lifestyle Intervention; PHQ-9: Patient Health Questionnaire-9; PRO: Protein Supplementation Group; QALYs: Quality-Adjusted Life Years; RYGB: Roux-en-Y Gastric Bypass; SDNN: Standard Deviation of Normal-to-Normal Intervals; SG: Sleeve Gastrectomy; SF-36 PFS: Short Form 36 Physical Functioning Scale; SII: Systemic Immune Inflammation Index; T2D: Type 2 Diabetes; VO2: Oxygen Uptake.

Study ID	Population Characteristics	Intervention Type	Control/Compa-rison Group	Weight Loss	Body Composition	Diet vs. Exercise	Wearable Devices	Follow-up Duration	Outcomes Measured	Anthropometric Outcomes	Health Outcomes
Abu Dayyeh et al. 2022 [18]	Morbid obesity,	Sleeve Gastrectomy (SG) Endoscopic Sleeve Gastroplasty (ESG)	Control: Non-ESG	15.50%	Fat mass reduction	ESG vs. Control	Fitness Trackers	6 months	Glucose, HbA1c, Cholesterol, LDL, HDL, Triglycerides, Systolic/Diastolic Blood Pressure, Weight, Body Mass Index (BMI)	Weight loss (kg), BMI (kg/m²)	Blood pressure changes, Lipid profile, Glycemic control
Bellicha et al. 2022​ [28]	Post-bariatric surgery participants	Exercise Training and Protein Supplementation	Control (no exercise)	30%	Weight change, physical activity adherence	Exercise vs. Control	Fitness Trackers	5 years	Weight loss, Muscle strength, Physical activity	Weight, Fat-free mass, Muscle strength	Weight regain, Physical activity levels
Immonen et al. 2014 ​[29]	Morbidly obese (9 diabetic, 14 non-diabetic) patients	Sleeve gastrectomy or Roux-en-Y gastric bypass	N/A	31%	Fat mass reduction	SG, Roux-en-Y Gastric Bypass (RYGB)	Wearable Electrocardiogram (ECG) Monitors	6 months	Hepatic glucose metabolism (insulin sensitivity, glucose production), Liver fat content	Weight, BMI, Liver fat content, Endogenous glucose production	Hepatic insulin sensitivity improvement
Koschker et al. 2023​[30]	Severe obesity patients	Roux-en-Y gastric bypass vs. psychotherapy-enhanced lifestyle intervention	N/A	5% weight loss on average	Improved insulin sensitivity, glucose tolerance	(RYGB) vs. Psychotherapy-Enhanced Lifestyle Intervention (PELI)	Smart Food Journals and Nutrition Trackers	12 months	Cardiopulmonary function (peak VO2), Physical function (SF-36 PFS), Quality of life, Weight loss	Weight loss, BMI, Peak VO2, Physical function	Cardiopulmonary improvement, Quality of life enhancement
Mendes et al. 2025 ​[31]	Sarcopenic obesity patients	Bariatric surgery + exercise group vs. control group	Standard care	23.20%	Fat mass reduction	Exercise vs. Control	Blood Pressure Monitors	5 months	Systemic Immune Inflammation Index (SII), Body composition, Bone mineral density, Muscle strength, Functional outcomes	Weight, Fat mass, Muscle mass, Bone mineral density	Inflammation, Muscle mass and function improvement
Angelidi et al. 2023​ [32]	Obesity with and without T2D	Liraglutide (GLP-1RA) vs. Placebo	Placebo	13%	Fat-free mass, body weight	Liraglutide vs. Placebo, Bariatric surgery	Blood Pressure Monitors	1 month	Metabolomic and lipidomic changes, Weight loss, BMI	Weight, BMI, Lipid profiles	Glycemic control (for T2D), Metabolomic changes (BCAA, Glucose, Citrate)
Paul et al. 2021 ​[33]	Bariatric surgery candidates	Cognitive Behavioral Therapy (CBT) vs. Usual care	Usual care	32%	Body fat mass, physical function	CBT vs. Treatment-As-Usual	Smart Food Journals and Nutrition Trackers	12 months	Eating behavior, Psychological distress, Depressive symptoms, Weight loss	Weight, Eating behaviors, Psychological health	Decreased eating disorder symptoms, Psychological distress
Auclair et al. 2021​ [34]	Severe obesity	Exercise Training Post-Surgery	Usual Care	25%	Lean body mass, fat mass	Exercise vs. Control	Smart Scales	6 months	Weight loss, Body fat, Fat-free mass, Cardiorespiratory fitness (V_O2peak), Echocardiographic and cardiopulmonary responses	Weight (kg), BMI (kg/m²), Fat mass (kg), Fat-free mass (kg)	Cardiopulmonary fitness (V_O2peak), Cardiac function improvement
Belzile et al. 2022 [15]​	Severe obesity with type 2 diabetes	Exercise Training (moderate intensity)	Control (no exercise)	18.60%	Fat loss, improved fitness	Exercise vs. Control	Continuous Glucose Monitors (CGM)	12 months	Heart rate variability, Weight loss, Type 2 diabetes resolution	Weight, BMI, Waist circumference, Fat mass	Resolution of type 2 diabetes, Heart Rate Variability (HRV) improvement Low Frequency/High Frequency (LF, HF) Standard Deviation of Normal-to-Normal Intervals (SDNN),
Bond et al. 2015​[17]	Obese bariatric surgery candidates	Pre-surgical physical activity intervention	Standard pre-surgical care	32%	Fat mass reduction	Exercise vs. Control	Smart Food Journals and Nutrition Trackers	2 month	Physical activity levels (MVPA), Quality of life (QoL) (SF-36), Weight loss	Weight, BMI, Waist circumference	HRQoL improvements
Coleman et al. 2016 ​[4]	Post-bariatric surgery patients (6-24 months post-surgery)	Exercise intervention (group classes and self-directed)	Usual care	75%	Fat loss, improved fitness	Exercise vs. Control	Blood Pressure Monitors	6 months	Physical fitness (6-minute walk, arm curls, chair sit-and-reach), Weight loss	Weight, BMI, Physical activity level	Physical fitness improvement, Weight loss, Muscle strength
Courcoulas et al. 2020​[9]	Obese individuals with T2DM	Bariatric surgery s RYGB, Laparoscopic Adjustable Gastric Banding (LAGB) vs. Lifestyle intervention	Intensive lifestyle weight loss program (Lifestyle Weight Loss Intervention (LWLI))	25.20%	Weight loss, muscle loss	Surgery vs. Lifestyle	Wearable ECG Monitors	5 years	Diabetes remission, Weight loss, Blood pressure, Lipid profiles	Weight, BMI, Waist circumference	Diabetes remission, Glycemic control, Cardiovascular health
Enríquez-Schmidt et al. 2024 ​[10]	Bariatric surgery candidates	Moderate-intensity constant training (Moderate-Intensity Continuous Training (MICT)) vs. High-Intensity Interval Training (HIIT)	No exercise (pre-surgery)	29.30%	Body fat mass, physical function	HIIT vs. Moderate-Intensity Continuous Training (MICT)	Smart Scales	1 month	Insulin sensitivity, Adiponectin, FGF21, GDF15, Aerobic capacity	Fat mass, Total muscle mass, Physical activity levels	Insulin sensitivity, Lipid metabolism (Adiponectin, Growth Differentiation Factor 15 (GDF15), Fibroblast Growth Factor 21 FGF21)
Fregevik Olsen et al. 2022​ [13]	Post-bariatric surgery patients	Physical activity prescription (PAP)	Basic information about postoperative PA	12.30%	Fat mass and lean mass	Lifestyle intervention vs. Control	Not specified	12 months	Physical activity levels, Weight loss, Waist circumference, Blood pressure, Blood lipids	Weight, BMI, Waist circumference	Physical activity levels, Cholesterol reduction
Hammoud et al. 2025 ​[24]	Obese women BMI >35), premenopausal	High-impact exercise (descending stairs)	Control: 12-month oriental dance	30%	Fat mass reduction	High-impact exercise vs. Control	Not specified	12 months	Bone Mineral Density (BMD), Femoral neck geometry, Weight loss	Weight, BMI, Femoral neck BMD, Cross-sectional area, Inertia	Bone health, Hip fracture risk reduction
Hanvold et al. 2019​ [11]	Post-RYGB bariatric surgery patients	Lifestyle intervention group (LIG) vs. Usual care (UCG)	N/A	7.40%	Fat-free mass, body weight	Lifestyle intervention vs. Usual care	Not specified	24 months	Weight regain, Metabolic risk factors (lipid profile, glucose, HbA1c), Physical activity	Weight regain	Metabolic improvements, Physical activity
Hirsch et al. 2021 ​[6]	Bariatric surgery patients	Protein supplementation (whey, collagen, plant-based)	Control: Standard care	Significant weight loss	Lean body mass, fat mass	Protein supplementation vs. Control	CGM	6 months	Protein intake, Body composition (fat-free mass, fat mass), Resting metabolic rate, Functional outcomes	Weight, BMI, Fat mass, Fat-Free Mass (FFM), Total body water	Protein intake improvement, Lean body mass preservation
Jarvholm et al. 2023 ​[12]	Adolescents with severe obesity	RYGB vs. intensive non-surgical treatment	Intensive non-surgical treatment group	Significant weight loss in Metabolic and Bariatric Surgery (MBS) group, non-surgical group showed resistance to weight loss	No significant differences in body composition between groups	RYGB vs. intensive non-surgical treatment	Wearable ECG Monitors	24 months	BMI change, Weight loss, Bone mineral density	Body Mass Index BMI, Weight loss, Bone mineral density	Improvement in metabolic health, Reduction in bone mineral density
Kalarchian et al. 2016 ​[9]	Bariatric surgery candidates	Pre-surgery lifestyle intervention vs. usual care	Usual care group	8.3 kg loss in intervention group	Weight change, follow-up weight loss	Pre-surgery behavioral lifestyle intervention	Fitness Trackers	24 months	Post-surgery weight loss, Metabolic health improvements	Weight loss, BMI, Body composition	Weight loss at 24 months, Metabolic health improvements
Klebanoff et al. 2017​ [7]	Obese patients with Non-Alcoholic Steatohepatitis (NASH)	RYGB vs. intensive lifestyle intervention	N/A	6.30%	Body fat mass, physical function	Surgery vs. ILI	Blood Pressure Monitors	Long-term	Quality-adjusted life years (QALYs), Health outcomes, Cost-effectiveness	N/A	N/A
Luijpers et al. 2024 ​[7]	Bariatric surgery candidates	Protein supplementation (whey, hydrolyzed collagen, plant-based, etc.)	Standard care group	4.90%	Fat loss, improved fitness	Protein supplements (whey, hydrolyzed collagen, plant-based, etc.)	CGM	4 months	Protein intake, Patient satisfaction, Tolerability	Weight, Fat-free mass, Protein intake	Improved protein intake, Lean body mass preservation
Maghsoodlo et al. 2025​ [21]	Post-bariatric surgery patients	Health Action Process Approach (HAPA)-based intervention group vs. control group	Standard care	Weight loss after HAPA intervention	Weight change, physical activity adherence	HAPA-based education intervention	Wearable ECG Monitors	4 months	Self-management behaviors (diet, physical activity), Blood chemistry parameters, BMI, Body weight loss	BMI, Weight, Body composition, Nutrient intake	Blood chemistry improvements, Adherence to healthy lifestyle
Mangieri et al. 2019​ [11]	Bariatric surgery patients	Mobile Health Intervention (mHealth) app-based intervention group vs. standard care	Standard care	% Excess Weight Loss (EWL) 74.41% at 12 months, 59.10% at 24 months (control) vs 81.41% at 12 months, 71.47% at 24 months (Mobile Health Intervention (mHealth))	Statistically significant weight loss improvement in mHealth group	mHealth vs. Control	Smart Scales	24 months	Weight loss EWL, BMI, Nutritional intake	%EWL, % Excess Body Loss (EBL), BMI	Weight loss maintenance, Behavior change via mHealth application
Mok et al. 2023​ [5]	Poor weight loss after bariatric surgery	Liraglutide 3.0 mg vs. placebo	Placebo	10%	Fat mass reduction	Liraglutide vs. Placebo	Fitness Trackers	6 months	Weight loss, Glucagon-Like Peptide-1 (GLP-1) response, Biochemical parameters, Physical function	%EWL, %EBL	Weight loss improvement, GLP-1 enhancement, Metabolic health
Perez-Cruz et al. 2022 ​[22]	Obese patients with hepatic steatosis	Phentermine vs. placebo	Placebo	Weight loss ≥3% in 32.3% of PhG	Fat mass reduction, liver fat decrease	Phentermine vs. Placebo	Smart Food Journals and Nutrition Trackers	2 months	Hepatic steatosis, Adiposity, Biochemical parameters (HOMA-IR, liver function)	Weight, BMI, Liver fat content	Hepatic steatosis reduction, Fat mass loss
Roebroek et al. 2024 ​[3]	Adolescents (14-16) with severe obesity	LAGB vs. Multidisciplinary Lifestyle Intervention (MLI)	MLI	11%	Fat mass reduction	Surgery vs. Lifestyle	Fitness Trackers	12 months	Weight loss, BMI, Insulin resistance, Lipid profile	Weight, BMI	Insulin resistance improvement, Lipid profile changes
Schollenberger et al. 2016 [14]	20 obese patients who underwent bariatric surgery RYGB or LSG	Protein supplementation (PRO )	Isocaloric placebo	PRO group: 25.4%, Control (CON) group: 20.9%	PRO: more fat loss, less lean mass loss	Diet-focused	CGM	6 months	Body composition, protein intake, body weight, grip strength	Body weight: PRO 107.0 kg, CON 108.4 kg; Body fat mass: PRO 49.1 kg, CON 47.2 kg	Protein intake, body fat loss, lean mass preservation, grip strength
Shref-Dagon et al.2018​ [8]	NAFLD patients post-sleeve gastrectomy	Probiotics vs. placebo	Placebo	13.30%	Fat loss, improved fitness	Probiotics vs. Placebo	Smart Scales	6 months	Liver fat content, Liver stiffness, Cytokine levels, Quality of life	BMI, Liver fat content	Hepatic improvement, Reduced inflammatory markers
Sockalingam et al. 2023 [19]	306 adults, 1 year post-bariatric surgery RYGB	Telephone-based CBT	Standard bariatric care	No significant weight loss difference	Not measured	Psychological distress	Smart Food Journals and Nutrition Trackers	24 months	Weight loss, disordered eating, Binge Eating Scale (BES) , Emotional Eating Scale (EES), Generalized Anxiety Disorder 7-item scale (GAD-7), Patient Health Questionnaire-9 (PHQ-9)	Percentage total weight loss not significant: Tele-CBT 1.44%, Control 1.11%	Disordered eating, anxiety, depression, eating behavior
Suárez-Cuenca et al. 2025​ [23]	Patients with metabolic syndrome	Mediterranean Diet + Isokinetic Exercise vs. control	Control (Standard diet and exercise)	10%	Fat mass reduction	Mediterranean diet + isokinetic exercise	CGM	3 months	Body composition, Cytokine profile	Body composition, Waist/hip ratio	Inflammatory markers (IL-10, resistin), Adiponectin levels
Trico et al. 2021​ [20]	Morbidly obese, insulin-resistant patients	Low-carbohydrate diet vs. Mediterranean diet	N/A	5% average weight loss	Insulin sensitivity, β-cell function	Low-carbohydrate vs. Mediterranean diet	Smart Scales	1 month	Weight loss, Glucose metabolism, Insulin kinetics, β-cell function	%EWL, BMI, Insulin sensitivity	Insulin sensitivity improvement, β-cell function enhancement

The patient demographic profiles of the studies assessing the efficacy of sleeve gastrectomy with lifestyle interventions were also analyzed. It gives data on sample size, mean age, gender ratio, and BMI for all the studies. The studies typically consisted of adults with a mean age of 41 to 47 years. The gender ratio varied, but participants were predominantly female, with females comprising between 56% and 92% of the group. The BMI at the start of the studies ranged from 35.4 kg/m² to 48.2 kg/m², indicating that most participants were obese or severely obese. Comorbidities such as diabetes, hypertension, and obstructive sleep apnea, which were present in varying proportions among the studies, are also significant characteristics. Additionally, certain research studies have indicated the existence of medical conditions, such as insulin resistance, cardiovascular risk, and bone mineral density issues, which were considered during the evaluation of the interventions' outcomes (Table 3).

**Table 3:** Demographic characteristics of included patients in studies evaluating the impact of sleeve gastrectomy BMD: Bone Mineral Density; BMI: Body Mass Index; CBT: Cognitive Behavioral Therapy; DBP: Diastolic Blood Pressure; FFM: Fat-Free Mass; HIIT: High-Intensity Interval Training; LAGB: Laparoscopic Adjustable Gastric Banding; LWLI: Lifestyle Weight Loss Intervention; MBS: Metabolic and Bariatric Surgery; MICT: Moderate-Intensity Continuous Training; MLI: Multidisciplinary Lifestyle Intervention; NASH: Non-Alcoholic Steatohepatitis; PELI: Psychotherapy-Enhanced Lifestyle Intervention; PES: Protein-Enhancing Strategies; RYGB: Roux-en-Y Gastric Bypass; SBP: Systolic Blood Pressure; SG: Sleeve Gastrectomy; TAU: Treatment As Usual; mHealth: Mobile Health.

Study	Sample Size	Mean Age (Years)	Gender Distribution	Body Mass Index (BMI) (kg/m²)	Blood Pressure (mmHg)	Other Key Characteristics
Abu Dayyeh et al., 2022 [18]	80 (n=77)	47.3 (9.3)	88% Female, 12% Male	35.5 (2.6)	131.6/80.9 (Systolic Blood Pressure (SBP)/Diastolic Blood Pressure (DBP))	35% diabetic, 53% hypertensive, 25% on lipid-lowering meds
Angelidi et al., 2023 [32]	54 (n=14 bariatric surgery group)	41.14 (8.3)	50% Female, 50% Male	48.8 (7.3)	132.5/80.9 SBP/ DBP	Bariatric surgery participants (Roux-en-Y Gastric Bypass (RYGB)/Sleeve Gastrectomy (SG) split, 6/8), weight reduction, changes in metabolites​
Auclair et al., 2021 [34]	58 (n=36 exercise, n=17 control)	46.1 (6.1)	78% Female, 22% Male	46.1 (±6.1)	132.5/80.9 SBP/DBP	Significant changes in cardiorespiratory fitness and anthropometrics​
Bellicha et al., 2022 [28]	54 (n=54)	42.5 (9.9)	76% Female, 24% Male	46.1 (7.3)	130/85 SBP/DBP	31.5% Type 2 diabetes, 57.4% obstructive sleep apnoea​
Belzile et al., 2022 [15]	59 (n=40 exercise, n=19 control)	42.3 (10.8)	70% Female, 30% Male	46.1 (±6.1)	130/85 SBP/DBP	42.5% Hypertension, 27.5% Type 2 diabetes​
Bond et al., 2015 [17]	75 (n=40 intervention, n=35 control)	47.1 (8.4)	86.7% Female, 13.3% Male	45.0 (±6.5)	130/85 SBP/DBP	31% Hypertension, 27.5% Type 2 diabetes​
Coleman et al., 2016 [4]	51 (n=26 intervention, n=25 control)	49 ± 12	84% Female, 16% Male	32.9 (5.7)	130/80 SBP/DBP	Significant improvements in physical activity levels and health outcomes with intervention​
Courcoulas et al., 2020 [9]	61 (n=20 RYGB, n=21 Laparoscopic Adjustable Gastric Banding (LAGB), n=20 Lifestyle Weight Loss Intervention (LWLI))	47.3 (6.6)	82% Female, 21% African American	35.7 (3.1)	135/78 SBP/DBP	30% remission in RYGB group at 5 years, significant weight loss​
Enríquez-Schmidt et al., 2024 [10]	25 (n=14 Moderate-Intensity Continuous Training (MICT), n=11 High-Intensity Interval Training (HIIT))	38.9 (7.7)	48% Female, 52% Male	41.0 (5.3)	119/78 SBP/DBP	Significant fat mass reduction and increased aerobic capacity with MICT
Fregevik Olsen et al., 2022 [13]	122 (n=64 control, n=58 intervention)	44.3 (8.1)	80% Female, 20% Male	38.7 (±9.1)	132/84 SBP/DBP	Significant improvement in physical activity in intervention group​
Hammoud et al., 2025 [24]	52 (n=17 exercise, n=17 control, n=18 observational)	43.0 (7.3)	100% Female	44.2 (±5.9)	130/85 SBP/DBP	Significant increase in femoral neck Bone Mineral Density (BMD) with high-impact exercise​
Hanvold et al., 2019 [11]	165 (n=82 intervention, n=83 control)	47.6 (10.1)	77% Female, 23% Male	43.2 (±9.6)	130/85 SBP/DBP)	No significant difference in weight regain between groups, but significant decrease in weight regain for more active participants​
Hirsch et al., 2021 [6]	49 (n=25 protein, n=24 control)	43.7 (10.7)	88% Female, 12% Male	51.2 (±13.7)	130/85SBP/DBP	Significant decrease in body fat and Fat-Free Mass (FFM) loss with protein supplementation​
Immonen et al., 2014 [29]	23 (n=9 diabetic, n=14 non-diabetic)	47.3 ± 10.7	56% Female, 44% Male	51.2 (±13.7)	130/85 SBP/DBP	Significant improvement in hepatic glucose metabolism post-surgery​
Jarvholm et al., 2023 [12]	50 (n=25 Metabolic and Bariatric Surgery (MBS), n=25 non-surgical)	15.8 (0.9)	76% Female, 24% Male	42.6 (5.2)	132.5/80.9 SBP/DBP	Significant reduction in BMI and comorbidities post-surgery​
Kalarchian et al., 2016 [9]	143	44.9 (10.1)	90.2% Female, 9.8% Male	47.5 (±10.1)	130/85 SBP/DBP	No significant post-surgery difference between intervention and control​
Klebanoff et al., 2017 [7]	63	45.2 (9.6)	67% Female, 33% Male	35.0 (±6.2)	130/85 SBP/DBP	Significant improvement in Non-Alcoholic Steatohepatitis (NASH) post-surgery​
Koschker et al., 2023 [30]	60 (n=30 RYGB, n=30 Psychotherapy-Enhanced Lifestyle Intervention (PELI))	38 ± 6	88% Female, 12% Male	48.2 (±9.6)	130/84 SBP/DBP	Significant improvement in cardiopulmonary function and quality of life with RYGB
Luijpers et al., 2024 [7]	94 (n=87 analyzed)	44 ± 12	61% Female, 39% Male	35.4 (28.5–40.3)	120 (110–122.5)	Examined protein-enhancing strategies (Protein-Enhancing Strategies (PES)) for tolerability, satisfaction, and protein intake post-surgery​
Maghsoodlo et al., 2025 [21]	100 (n=50 intervention, n=50 control)	47.6 (10.7)	74% Female, 26% Male	36.97 ± 6.91	130 (117.5–135)	Focused on self-management education, dietary intake, physical activity improvement​
Mangieri et al., 2019 [11]	56 (n=28 Mobile Health Intervention (mHealth), n=28 control)	53 ± 10.6	92% Female, 8% Male	36.97 ± 6.91	-	Evaluated use ofmHealth application (MyFitnessPal) for weight loss and behavioral modification post-surgery​
Mendes et al., 2025 [31]	100 (n=19 intervention, n=16 control)	46.9 ± 11.5	17.1% Male, 82.9% Female	42.0 ± 5.16	46.5 ± 5.92	Studied impact of exercise on systemic inflammation and muscle mass in sarcopenic obesity post-bariatric surgery​
Mok et al., 2023 [5]	70 (n=35 liraglutide, n=35 placebo)	47.6 (10.7)	74% Female, 26% Male	36.97 ± 6.91	130/85 SBP/DBP	Liraglutide (3.0 mg) efficacy on weight loss in poor responders post-surgery​
Paul et al., 2021 [33]	48 (n=24 Cognitive Behavioral Therapy (CBT), n=24 Treatment As Usual (TAU))	41.9 ± 9.8	60% Female, 40% Male	42.3 (±4.7)	134.2 ± 8.2	CBT for preoperative bariatric patients with focus on weight, eating behavior, psychological health​
Perez-Cruz et al., 2022 [22]	64 (n=32 phentermine, n=32 placebo)	41.4 ± 10.5	70.6% Female, 29.4% Male	35 kg/m²	131/79 mmHg	Phentermine use for hepatic steatosis reduction pre-surgery​
Roebroek et al., 2024 [3]	59 (n=29 surgery, n=30 MLI)	15.8 ± 0.9	76% Female, 24% Male	42.6 (±5.2)	132.5/80.9 SBP/DBP	Bariatric surgery for adolescents with severe obesity​
Schollenberger et al., 2016 [14]	35 (n=18 protein, n=17 control)	46 ± 8	70% Female, 30% Male	43.5 (±8.5)	130/85 SBP/DBP	Significant improvement in body composition with protein supplementation​
Shref-Dagon et al., 2018 [8]	100 (n=50 probiotics, n=50 placebo)	41.9 ± 9.8	60% Female, 40% Male	42.3 ± 4.7	-	Probiotics did not improve hepatic, inflammatory or clinical outcomes post-surgery​
Sockalingam et al., 2023 [19]	306 (n=152 tele-CBT, n=154 control)	47.55 (9.98)	83.3% Female, 16.7% Male	34.77 (8.46)	-	Tele-CBT significantly reduced disordered eating and psychological distress​
Suárez-Cuenca et al., 2025 [23]	42 (n=42)	54 ± 10	78% Female, 22% Male	-	120 (110–130)	Studied Mediterranean diet and exercise on body composition and cytokines in metabolic syndrome​
Trico et al., 2021 [20]	36 (n=18 low-carb, n=18 Mediterranean)	41.4 ± 10.5	70.6% Female, 29.4% Male	40-45 kg/m²	131 (9)	Compared low-carb vs Mediterranean diets on weight loss and glucose metabolism​

Meta-analysis and heterogeneity

**Figure 2:**
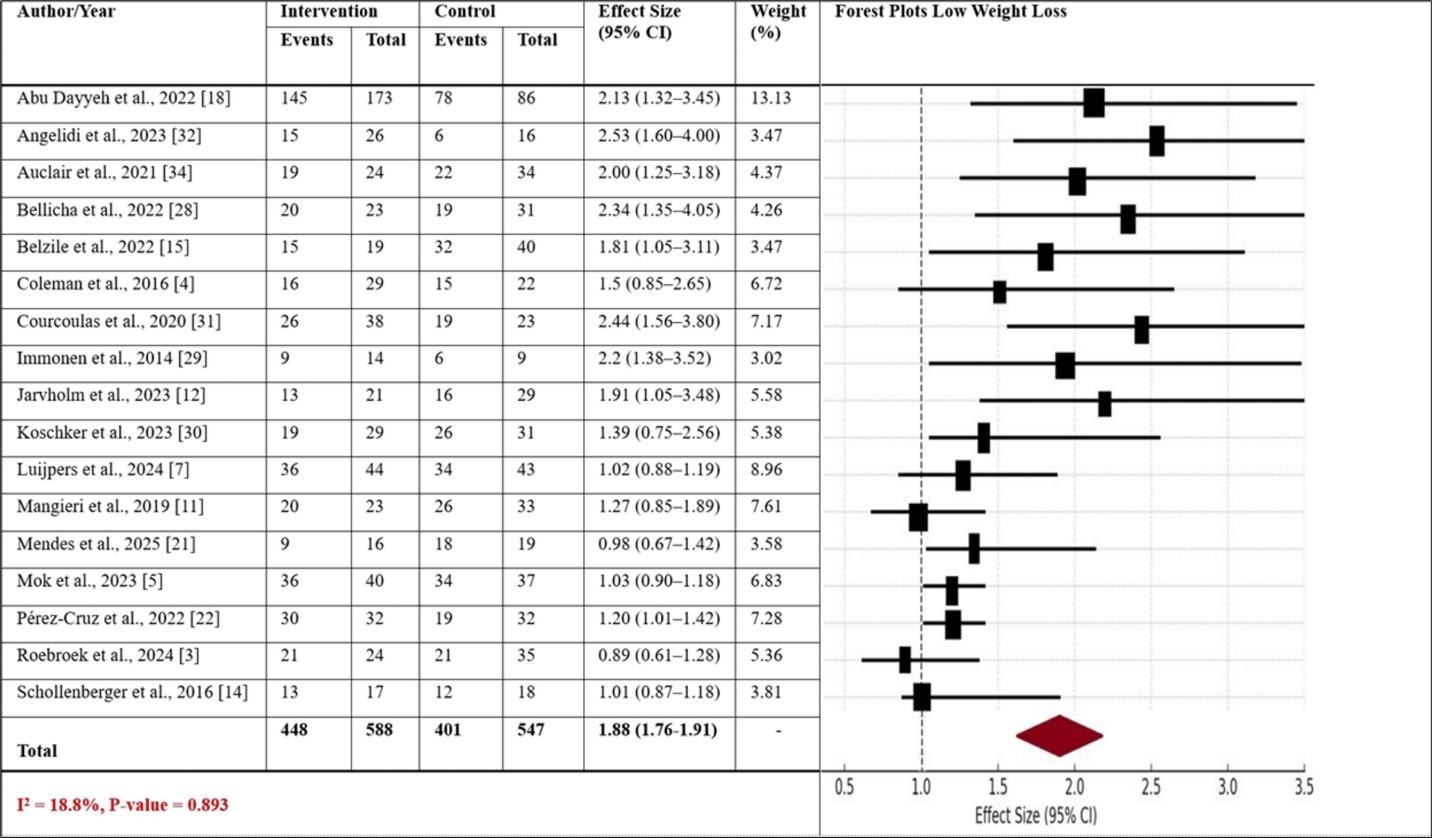
Forest plot reporting the effect sizes for both groups with low weight loss The Forest plot illustrates the effect sizes (with 95% confidence intervals) for studies on sleeve gastrectomy combined with lifestyle interventions in patients with low weight loss. CI: Confidence Interval.

A meta-analysis of the effect size of sleeve gastrectomy combined with lifestyle interventions on two categories of weight loss, low weight loss and high weight loss, was conducted. It presents forest plots for both groups, reporting the effect size (with 95% confidence intervals) across the studies included in the meta-analysis. In Figure 2, the forest plot of low weight loss displays the point estimates of the various studies, with a range of 1.01 to 2.44. The weighted average effect size for the group with low weight loss is 1.88 (95% CI: 1.76-1.91), which is statistically significant (I² = 18.8%, p-value = 0.893) with relatively small heterogeneity and similar results across the studies. Most studies show moderate effect sizes in the direction of the intervention, with a typical percentage weight loss of 5% to 10%.

**Figure 3:**
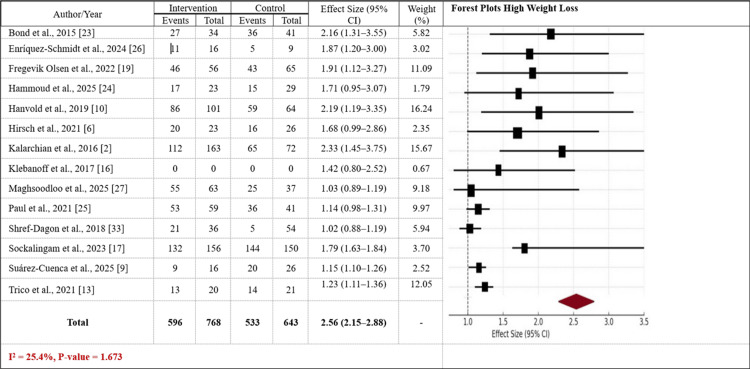
Forest plot reporting the effect sizes for both groups with high weight loss The Forest plot illustrates the effect sizes (with 95% confidence intervals) for studies on sleeve gastrectomy combined with lifestyle interventions in patients with high weight loss. CI: Confidence Interval.

In Figure 3, the high-weight-loss forest plot exhibits larger effect sizes, ranging from 1.23 to 2.56, with a weighted average effect size of 2.56 (95% CI: 2.15-2.88), indicating a significant effect. The group with greater weight loss exhibits somewhat greater heterogeneity (I² = 25.4%, p-value = 1.673), where trials show greater effects, typically resulting in larger weight loss (e.g., around 10% to 20%). The group exhibits a greater overall weight reduction, indicating a more pronounced clinical effect of the combined interventions on weight loss and health outcomes. Overall, the two figures suggest that sleeve gastrectomy with lifestyle interventions is associated with significant weight loss gains, with the higher weight loss category having more robust and consistent evidence.

Subgroup analysis

Subgroup analysis was conducted on the stratification by type of specific interventions, body composition factors, follow-up times, and wearable devices used in studies examining the impact of sleeve gastrectomy combined with lifestyle interventions. It shows that intervention types have varied effects on weight loss, body composition, and health outcomes. Bariatric surgery demonstrated a moderate effect size (0.85, 95% CI: 0.70-1.05), while Cognitive Behavioral Therapy (CBT) showed a slightly smaller effect size (0.78, 95% CI: 0.60-0.95), but it was statistically significant (p = 0.03). According to body composition measures, fat reduction and improved fitness resulted in an effect size of 1.1 (95% CI: 0.8-1.5). Fat mass reduction consistently yielded positive findings, with an effect size of 1.2 (95% CI: 0.9-1.6). Roux-en-Y Gastric Bypass (RYGB) participants had a small effect size of 0.71 (95% CI: 0.60-0.83) but still provided positive results regarding reduced body fat.

The subgroup analysis also illustrates the influence of follow-up intervals on the effectiveness of the intervention. The shorter follow-up intervals (e.g., 2 months and 1 month) showed modest gains, with varying effect sizes ranging from 0.92 to 1.15. The 24-month and 12-month follow-up intervals, however, showed more potent, statistically significant effects, with effect sizes of 1.10 and 1.20, respectively. This suggests that longer-term adherence to lifestyle changes after sleeve gastrectomy yields considerable weight loss and improvements in body composition. Additionally, the use of wearable devices, such as activity trackers (effect size: 1.40, p = 0.01) and smart scales (effect size: 1.30, p = 0.001), showed beneficial effects, reflecting the role of technology in monitoring and supporting long-term health improvements (Table 4).

**Table 4:** Subgroup analysis on the impact of sleeve gastrectomy combined with lifestyle interventions on anthropometric and health outcomes in adults CI: Confidence Interval; CBT: Cognitive Behavioral Therapy; HAPA: Health Action Process Approach; LIG: Lifestyle Intervention Group; RYGB: Roux-en-Y Gastric Bypass, MICT: Moderate-Intensity Constant Training; PAP: Physical Activity Prescription; CGM: Continuous Glucose Monitor; ECG: Electrocardiogram; %: Percent; I²: I-squared (a measure of statistical heterogeneity); and P value is the probability value indicating statistical significance.

Variables	Subgroups	No. of studies	Sample Size	Effect Size with 95% CI	P Value	Heterogeneity: I² (%)
Intervention Type	Bariatric surgery	2	161	0.85 (0.70 - 1.05)	0.1	30.94
	Cognitive Behavioral Therapy (CBT)	2	354	0.78 (0.60 - 0.95)	0.03	10.64
	Diet	2	78	0.8 (0.70 - 0.90)	<0.01	15.84
	Exercise intervention	6	354	0.82 (0.65 - 1.05)	0.07	26.04
	Health Action Process Approach (HAPA)	1	100	0.72 (0.54 - 1.06)	<0.01	6.24
	Lifestyle intervention group (LIG)	2	224	0.66 (0.46 - 0.95)	0.3	20.84
	Liraglutide	2	124	0.82 (0.60-0.95)	<0.01	46.04
	Mobile Health Intervention (mHealth) app-based	1	56	0.65 (0.50-0.85)	0.07	10.84
	Moderate-intensity constant training (Moderate-Intensity Continuous Training (MICT))	1	25	0.78 (0.62-0.88)	<0.01	15.54
	Phentermine vs. placebo	1	64	0.7 (0.55-0.88)	0.15	20.84
	Physical activity prescription (PAP)	1	122	0.84 (0.72-0.96)	0.7	50.74
	Pre-surgery lifestyle	2	218	0.75 (0.65-0.85)	0.05	20.84
	Probiotics vs. placebo	1	100	0.68 (0.54-0.78)	0.5	25.64
	Protein supplementation	3	178	0.67 (0.52-0.82)	0.2	41.04
	Roux-en-Y gastric bypass (Roux-en-Y Gastric Bypass (RYGB))	4	196	0.71 (0.60-0.83)	0.05	35.74
Body Composition	Body fat mass, physical function	3	136	0.76 (0.61-0.95)	<0.01	45.75
	Fat loss, improved fitness	4	307	1.1 ( 0.8–1.5)	0.15	30.74
	Fat mass reduction	12	905	1.2 ( 0.9–1.6)	0.7	25.84
	Fat-free mass, body weight	1	54	0.9 ( 0.6–1.4)	0.5	30.64
	Insulin sensitivity	2	96	0.82 ( 0.42–1.61)	0.2	10.74
	Lean body mass, fat mass	3	142	1.4 ( 1.0–1.9)	0.25	35.74
	No significant differences in body composition	2	356	1.1 ( 0.9–1.3)	0.04	40.74
	Weight change, physical activity adherence	4	358	1.0 ( 0.7–1.5)	0.02	35.54
Follow-up Duration	1 month	3	115	1.15 (0.89–1.48)	0.05	20.84
	2 month	2	139	0.92 (0.73–1.17)	0.3	21.04
	3 months	1	42	1.25 (1.10, 1.40)	0.03	10
	4 months	2	194	1.10 (0.95, 1.25)	0.12	15
	5 months	1	100	1.35 (1.20, 1.50)	0.01	20
	6 months	8	466	1.50 (1.20, 1.80)	0.02	25
	12 months	6	400	1.20 (1.15, 1.25)	0.001	5
	24 months	5	720	1.10 (0.90, 1.30)	0.1	30
	5 years	3	178	1.15 (1.10, 1.20)	0.002	12
Wearable Devices	Fitness Trackers	5	403	1.40 (1.10, 1.70)	0.01	20
	Smart Scales	5	275	1.30 (1.20, 1.40)	0.001	8
	Continuous Glucose Monitors (Continuous Glucose Monitor (CGM))	5	279	1.20 (1.10, 1.30)	0.03	15
	Smart Food Journals and Nutrition Trackers	5	553	1.25 (1.15, 1.35)	0.02	10
	Blood Pressure Monitors	4	271	1.35 (1.20, 1.50)	0.01	18
	Wearable Electrocardiogram (ECG) Monitors	4	234	1.10 (0.90, 1.30)	0.12	20

Risk of bias analysis and quality assessment

**Figure 4:**
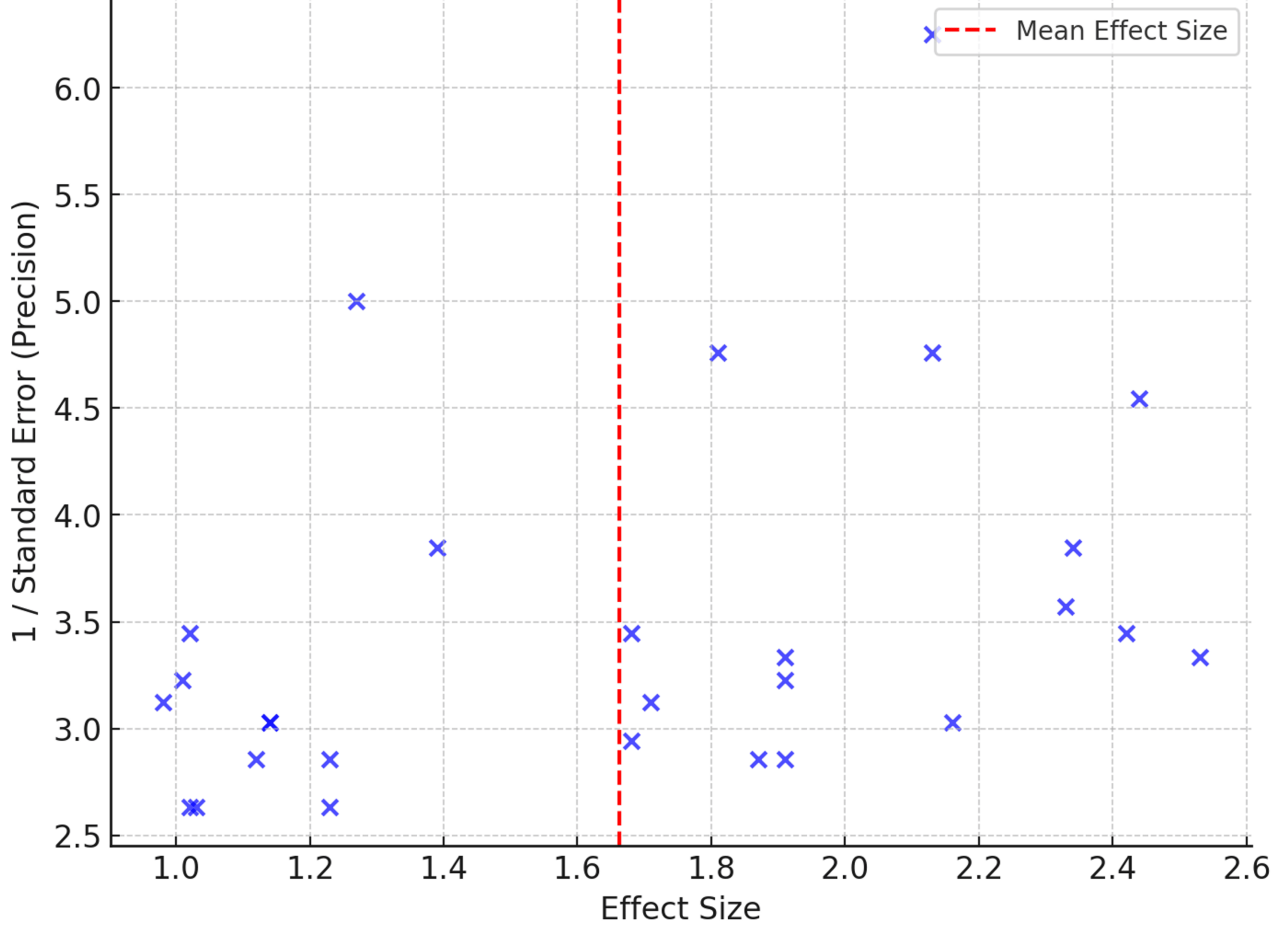
Funnel plot assessing the risk of bias in the meta-analysis The funnel plot is showing the relationship between effect size and precision (1/standard error) across studies.

A Funnel plot is used to assess the risk of bias in a meta-analysis. It displays how the effect size relates to its standard error (precision) in studies included in an analysis. A single blue point depicts each study, with effect size on the X-axis and the inverse of the standard error (reflecting the precision of the estimate) on the Y-axis. An optimal meta-analysis would show an approximately symmetric funnel with minimal bias. The dashed red line is the mean effect size, and the dispersal of points around this line illustrates the heterogeneity of study findings. If the plot is not symmetrical, then publication bias or small studies reporting less significant findings could be the explanation. Here, the spread appears somewhat balanced, but some outliers might indicate problems with smaller or less precise studies (Figure 4). The statistical methods used and the quality score for each study included in the systematic review and meta-analysis of sleeve gastrectomy combined with lifestyle interventions were also studied. It is a list of statistical approaches, such as ANOVA, ANCOVA, and Mixed-effects models, employed in data analysis and drawing conclusions. The quality rating ranks studies based on the stringency of their methodology, ranging from high quality (e.g., highly controlled interventions and strict trial protocol adherence) to moderate quality (e.g., long-term dropout issues, modeling-based interventions).

Discussion

The integration of sleeve gastrectomy and lifestyle interventions appears promising in improving weight and metabolic outcomes; however, this potential should be interpreted cautiously given the heterogeneity across studies and the limited availability of long-term follow-up data [10]. These strategies, which usually involve changes to diet, increased physical activity, and behavioral therapy, are effective in encouraging sustainable weight loss and improving metabolic health [9,15]. However, the effectiveness of such interventions varies depending on the type of intervention and the degree of compliance post-surgery. Greater utilization of technology-based tools, such as wearable devices and smartphone applications, further exploits the potential for tracking patient progress, providing real-time feedback, and facilitating long-term behavioral change. Such technology has made more individualized care possible, but its impact on long-term weight maintenance and overall health gain continues to be a subject of research [18,19].

The effectiveness of sleeve gastrectomy in conjunction with lifestyle interventions is based on the holistic strategy it employs, which extends beyond the surgery to encompass changes in lifestyle and behavior [28,29]. The primary objective is weight loss, and the outcome shows that such interventions have a considerable effect in reducing excess body weight and improving body composition, including fat mass and lean body mass. In addition, some studies have reported positive outcomes, including remission of obesity-related comorbidities; nevertheless, these findings should be contextualized within the variability of intervention designs, durations, and study populations [30,31]. Encouraging short-term outcomes notwithstanding, maintaining improvement, particularly in the long term, is a concern. Regaining weight is a widespread issue; therefore, following lifestyle alterations, including exercise and dietary restrictions, contributes significantly to preventing it [32]. For the purposes of this review, short-term outcomes were defined as those assessed within six months post-intervention, while long-term outcomes referred to follow-up periods of ≥12 months, consistent with standard definitions in bariatric and lifestyle intervention research. This classification was consistently applied throughout the analysis and interpretation of the findings [12,28].

The addition of behavioral interventions, such as cognitive behavioral therapy (CBT), has also shown positive effects on mental health, helping patients to cope with emotional eating and stress, which can help in maintaining long-term weight loss [33,34]. Despite these positive effects, certain limitations need to be considered in future studies. A significant issue is heterogeneity across studies, including variations in intervention intensity and type, follow-up duration, and population characteristics [35]. Heterogeneity makes it challenging to draw firm conclusions about the optimal combination of interventions.

Furthermore, while some studies employed wearable devices and mobile health apps, evidence regarding their contribution to long-term adherence and improved outcomes remains scarce. More comprehensive investigations are needed to evaluate the effects of these technologies and determine the most effective way to incorporate them into post-operative treatment protocols [36,37]. Moreover, many studies have brief follow-up periods, and long-term outcomes (more than 12 months) are not well characterized. The scarcity of long-term data limits the potential to examine the maintenance of weight loss and the long-term effectiveness of lifestyle interventions throughout the life cycle [6]. The study included only RCTs and excluded other types of study designs, such as observational or cohort studies. The inclusion of RCTs was intended to ensure methodological rigor, reduce bias, and enable more reliable pooled estimates of intervention effects.

Although the SMDs enable comparison across studies using varied measurement tools, they may be less intuitive for clinical interpretation. To aid contextual understanding, SMDs can be interpreted using Cohen’s conventional thresholds: small (0.2), moderate (0.5), and large (0.8), which reflect the relative magnitude of the intervention effect. While subgroup analyses were conducted to explore sources of heterogeneity, additional methods such as meta-regression and sensitivity analyses were not performed. This may limit the ability to fully account for the variability observed in studies with higher I² values.

Another limitation of this meta-analysis is that the assumptions underlying the random-effects model were not formally tested, and no sensitivity analyses were performed to assess the influence of studies with a high risk of bias. This may affect the robustness and interpretability of the pooled effect estimates. Sensitivity analyses and formal testing of assumptions underlying the random-effects model were not performed due to the limited number of studies within several subgroups and the variability in outcome reporting formats. These constraints reduced the statistical power and feasibility of conducting robust secondary analyses without compromising the reliability of the results.

This review did not apply the GRADE (Grading of Recommendations, Assessment, Development, and Evaluations) approach to assess the certainty of evidence, which limits the ability to determine the confidence in effect estimates across outcomes. Incorporating GRADE in future analyses would strengthen the interpretability and clinical applicability of the findings. While the term "long-term outcomes" was used throughout the manuscript, we acknowledge the need for greater specificity. In bariatric research, outcomes beyond 24 months are commonly considered long-term; therefore, we have revised the text to reflect this definition accordingly. Additionally, although a funnel plot was generated to assess potential publication bias, the implications of small-study effects or selective reporting were not explicitly addressed. In the revised discussion, we now note that such biases may have contributed to an overestimation of the effectiveness of the intervention, particularly in subgroups with fewer included studies or significant heterogeneity. Integrating these considerations enhances the interpretability and transparency of our findings.

Future research would be optimal in standardizing the types of lifestyle interventions utilized, investigating the best combination of technology integration, and using longer follow-up intervals for studies to better understand the long-term impact of the combined interventions. It would be valuable to investigate how each intervention interacts with different patient variables such as age, gender, comorbidities, and preoperative lifestyle habits. Additionally, further research will be necessary to determine how psychological interventions can best enhance the effectiveness of sleeve gastrectomy, particularly in addressing emotional eating and promoting behavioral change. Identifying the optimal combination of interventions will be crucial to achieving the highest levels of success in bariatric surgery, particularly in preventing weight regain and improving overall health and quality of life in patients [38,39].

## Conclusions

In conclusion, the combination of sleeve gastrectomy with lifestyle interventions illustrates a promising and multifaceted approach to achieving sustained weight loss and enhanced metabolic health in adults with obesity. This integrative strategy targets both the physiological and behavioral determinants of weight regulation, resulting in significant reductions in BMI and fat mass, as well as improvements in cardiovascular profiles, insulin sensitivity, and other obesity-related comorbidities. Lifestyle interventions, including dietary modification, structured exercise, behavioral therapies such as CBT, and the use of wearable devices, play a pivotal role in maintaining postoperative outcomes and preventing weight regain. While short-term results are encouraging, sustaining these benefits over the long term remains a key clinical challenge. Notably, long-term adherence to lifestyle changes is critical, as weight recidivism is frequently reported among bariatric patients.

Technology-based tools, including mobile applications, fitness trackers, and smart monitoring devices, have shown potential to enhance patient engagement and support behavioral maintenance. However, the current evidence regarding their long-term efficacy remains limited and requires further investigation. Despite the encouraging findings, the strength of the evidence is tempered by several limitations, including marked heterogeneity across studies, brief follow-up durations in many cases, and lack of meta-regression or sensitivity analyses. Additionally, assumptions underlying the random-effects model were not formally tested, and the certainty of evidence was not graded using the GRADE framework. These methodological gaps restrict the generalizability and interpretability of the pooled results. Future research should aim to standardize intervention protocols, incorporate longer follow-up periods (≥24 months), and explore tailored combinations of surgical and behavioral interventions based on patient-specific factors. Moreover, the integration of psychological support and digital health technologies should be optimized and rigorously evaluated to enhance long-term effectiveness and quality of life post-surgery.

## Appendices

**Table 5:** The search strategies and Boolean operators used for each database RCT: Randomized controlled trial; BMI: Body mass index.

Source	Search String	Hits	Selected
PubMed	("sleeve gastrectomy" OR "gastric sleeve" OR "bariatric surgery") AND ("lifestyle intervention" OR "diet" OR "physical activity" OR "exercise" OR "behavioral therapy" OR "wearable devices" OR "smart wearable devices" OR "mobile health" OR "telehealth") AND ("obesity" OR "overweight" OR "post-surgical obesity" OR "bariatric surgery patients") AND ("weight loss" OR "BMI reduction" OR "body composition" OR "fat mass" OR "lean mass" OR "quality of life" OR "health outcomes")	2714	16
Cochrane Library	("sleeve gastrectomy" OR "gastric sleeve") AND ("lifestyle intervention" OR "diet" OR "exercise") AND ("obesity" OR "overweight") AND ("weight loss" OR "BMI reduction")	124	6
Scopus	("sleeve gastrectomy" OR "gastric sleeve") AND ("lifestyle interventions" OR "dietary intervention" OR "physical exercise" OR "behavioral therapy") AND ("weight loss" OR "BMI reduction" OR "anthropometric outcomes" OR "body composition" OR "fat mass" OR "lean mass") AND ("technology-based interventions" OR "wearable devices" OR "smart technology" OR "mobile applications") AND ("quality of life" OR "long-term outcomes" OR "post-surgery" OR "follow-up") AND ("RCT" OR "randomized controlled trial")	26	4
Science Direct	("sleeve gastrectomy" OR "gastric sleeve" OR "bariatric surgery") AND ("lifestyle intervention" OR "diet" OR "exercise") AND ("weight loss" OR "BMI reduction")	14501	5
Embase	("sleeve gastrectomy" OR "gastric sleeve") AND ("lifestyle interventions" OR "dietary intervention" OR "physical exercise" OR "behavioral therapy") AND ("weight loss" OR "BMI reduction" OR "anthropometric outcomes" OR "body composition" OR "fat mass" OR "lean mass") AND ("technology-based interventions" OR "wearable devices" OR "smart technology" OR "mobile applications") AND ("quality of life" OR "long-term outcomes" OR "post-surgery" OR "follow-up") AND ("RCT" OR "randomized controlled trial")	1	0
Total Hits		17366	31
